# Urban-Rural Disparity in Cognitive Performance Among Older Chinese Adults: Explaining the Changes From 2008 to 2018

**DOI:** 10.3389/fpubh.2022.843608

**Published:** 2022-03-23

**Authors:** Tao Zhang, Beiyin Lu, Xiaohe Wang

**Affiliations:** Department of Health Policy and Management, School of Public Health, Hangzhou Normal University, Hangzhou, China

**Keywords:** cognitive performance, elderly, urban-rural disparity, dynamic decomposition, China

## Abstract

**Objectives:**

This study aims to identify the dynamic changes in cognitive performance differentials between urban and rural older adults in China from 2008 to 2018 and decomposes determinants affecting such changes.

**Methods:**

Two waves (2008 and 2018) of data were extracted from the Chinese Longitudinal Healthy Longevity Survey. The cognitive function was tested using the Chinese Mini-Mental State Examination (MMSE). The effects of the explanatory variables (demographic, economic, neighborhood, environmental events and social and cultural domains) on the changes in the urban-rural inequality of cognitive performance were divided into two components using the Juhn–Murphy–Pierce (JMP) decomposition: quantity effect and price effect.

**Results:**

A total of 14,628 (urban respondents: 5,675, rural respondents: 8,953) and 10,311 older adults (urban respondents: 5,879, rural respondents: 4,432) for 2008 and 2018, respectively, were included in our study. A narrowing of 0.071 in the urban-rural disparity in cognitive function score of the older adults from 2008 to 2018 was identified. Quantity and price effects of explanatory variables contributed 65.21 and 46.84%, respectively, to the observed components in explaining the narrowed disparity. Quantity effects of age (35.71%), exercise (56.72%), self-rated economic status (33.19%) and price effect of homeownership (54.97%) contributed significantly to the reduced urban-rural gap. Contrastingly, inequality in pension (−27.31%) and social security (−23.11%) between urban and rural widened cognitive performance differentials. Furthermore, effects of hunger in childhood (−10.53%) and less years of schooling (−77.20%) on the increase in urban-rural inequality seemed to be stronger over time.

**Conclusion:**

Economic development and reform of the rural health system are responsible for the decline in the urban-rural disparity in the cognitive performance of older adults. Equalizing the distribution of social security and welfare between urban and rural must be highlighted for eliminating cognitive ability disparity. Additionally, rural older adults who endured hunger and poor education in childhood also deserve further policy interventions.

## Introduction

Cognitive impairment is a syndrome that reduces mind or intellectual activity. Given that late life cognitive changes may not initially appear to directly affect daily living, the degeneration of cognitive function may be left undiagnosed or be diagnosed at later stages (1, 2). In recent years, the eye-catching prevalence of cognitive impairment has been expected to dramatically increase with rapid aging in China. Jia et al. reported a total of 38.77 million people aged 60 and more suffer from cognitive impairment, and the prevalence of cognitive impairment amongst older adults in China was 15.54% (3).

To make matters worse, the urban-rural inequality in cognitive health has been plaguing the Chinese government. Previous studies have demonstrated a widened urban-rural disparity in cognitive performance amongst older population in China. For example, Zhang et al. found that rural older adults suffered from more severe cognitive impairment due to inadequate access to healthcare (4). Sun et al. reported that urban older adults had better cognition than their rural counterparts (5). Tian et al. uncovered that rural older adults had worse psychological disorders compared with their urban counterpart (6). This cognitive disadvantage amongst rural residents is usually explained by the negative impacts of rural living, such as limited access to education, workforce participation and healthcare (7).

It is well known that China is a huge country with significant urban-rural differences in terms of social and economic circumstances. Large academic medical centers, tertiary hospitals and skilled medical practitioners are concentrated in urban areas, which resulted in the inequality of health services between rural older adults and their urban counterpart (8, 9). Limited health resources and unfavorable economic status hinder rural residents from receiving timely treatment for physical and mental illnesses (10, 11). Consequently, rural older adults suffer from disproportionately poorer health and worse health outcomes compared with urban older adults.

To eliminate the urban-rural disparity in healthcare, many strategies have been taken at the national level over the past decade. For example, healthcare resources were redistributed across rural areas since the new round of healthcare system reforms in 2009 (12). Urban Resident Basic Medical Insurance (URBMI) and New Cooperative Medical Scheme (NCMS) were integrated to reduce urban-rural inequality in access to health services (13). However, it remains unclear whether the gap between the cognitive health of urban and rural older adults is narrowing after these efforts, and which determinants contribute to such changes, if any, in rural-urban disparity in cognitive health. To our knowledge, no study has provided related empirical evidence to answer the above-mentioned questions, given that most studies only adopted a cross-sectional data, and time series econometric methods were rarely used.

This study aims to identify the dynamic changes in cognitive performance differentials between urban and rural older adults over a 10-year period (2008–2018) in China and further decomposes determinants affecting this change. To our knowledge, this is first study in China that focuses on the dynamic changes of urban-rural disparity in the cognitive ability of older adults. Findings of the study will shed some light on future priorities to further narrow the rural-urban disparity in health outcomes.

## Materials and Methods

### Data Source

Data used in this study were extracted from the Chinese Longitudinal Healthy Longevity Survey (CLHLS), a prospective cohort study on the health status of older adults (≥65 years) people in China and its social, behavioral and biological determinants. CLHLS started in 1998, and eight waves of surveys have been completed up to date. In each wave, those lost persons were recorded and new participants from the neighboring households were added to ensure the sample is nationally representative. During investigation, a multistage, stratified cluster sampling design was applied to recruit participants from 23 of the 31 provinces in China. CLHLS randomly selected 631 cities and counties representing roughly 85% of the Chinese population. The questionnaire for CLHLS includes many variables, such as basic information, health status, family status, lifestyle, healthcare services and so on. CLHLS provides high-quality and nationally representative information for studying the health status of older adults in China, and more details can be found elsewhere (14, 15).

In this study, two waves of data were selected to meet the needs of dynamic decomposition: the most recent one collected in 2018 was compared with the one collected in 2008. These two waves of data were chosen for the following reasons. First, China has experienced rapid urbanization and a series of social reform from 2008 to 2018. Decomposition using these two waves of data reflects the impact of social change. Second, data collected prior to 2008 in CLHLS does not include some variables (for example, trip, social services and social security) we need in this study.

Due to some samples containing missing values on the outcome and explanatory variables, 1,083 respondents in 2008 and 1063 respondents in 2018 were excluded for data analysis. After filtering the data, 14,628 for the 2008 cohort and 10,311 for the 2018 cohort were included in our study. The basic characteristics (including age, gender, marital status, economic status) of the respondents did not change significantly before and after the sample was screened.

### Outcome Variables

In CLHLS, the cognitive performance of older adults was assessed using the Chinese version of Mini Mental State Examination (MMSE), which has been proved to be reliable and valid for older Chinese adults (16, 17). MMSE contains 24 items from the five aspects of cognitive function: five items for orientation, three items for reaction, five items for attention and calculation, three items for recall and eight items for language. Correct answers were coded as 1, otherwise they were coded as 0. The total score of the MMSE ranges from 0 to 30 points, with higher scores indicating higher cognitive ability.

### Explanatory Variables

**Figure 1:**
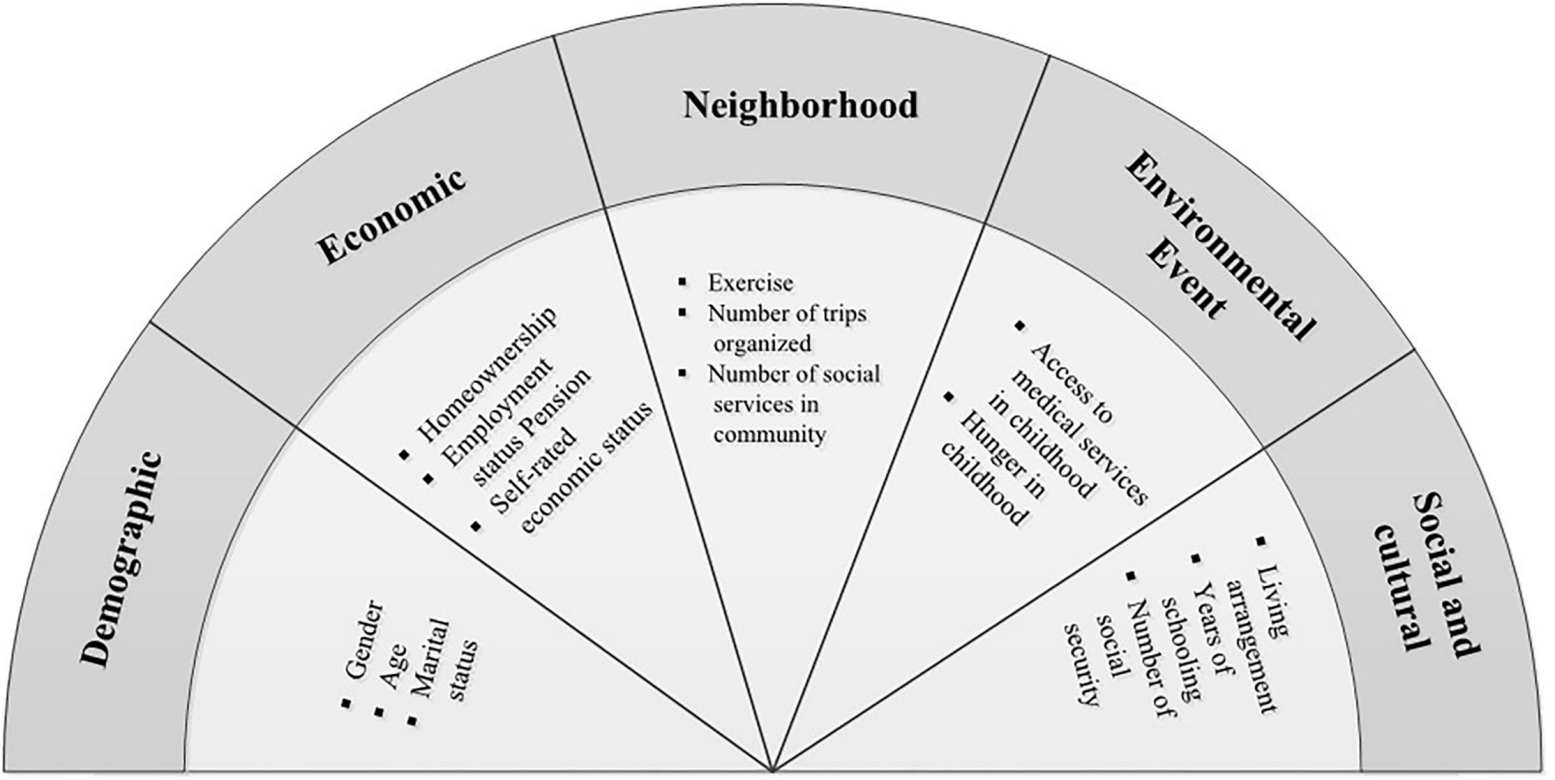
Variable construction by domain based on Lund's conceptual framework.

Following the conceptual framework developed by Lund et al. (18), 15 variables were selected from five domains: demographic, economic, neighborhood, environmental events and social and cultural domains (Figure 1). Domain definitions from Lund's study and variable construction are provided in Supplementary Table 1. These domain variables have provided a comprehensive picture to predict mental disorders and have been reported in the literature (18).

The demographic domain includes gender, age and marital status. The economic domain includes homeownership, employment status, pension and self-rated economic status. In the neighborhood domain, three variables were selected: regular exercise, number of trips organized and number of social services in the community. The environmental events contained two variables related to the respondent's experience of hunger and access to healthcare in childhood. Social and cultural domains include living arrangements, years of schooling and amount of social security and commercialized insurance.

### Statistical Analyses

Firstly, *t*-test and Pearson χ2 test were employed to compare the rural-urban differences in MMSE scores and explanatory variables, respectively. Then, to decompose the changes in rural-urban disparity in the cognitive performance of older adults from 2008 to 2018 and identify the contributions of each explanatory variable to such changes, a two-step approach was adopted: Oaxaca–Blinder (O–B) static decomposition and JMP dynamic decomposition.

### Step One: O–B Decomposition

On the basis of the O–B method, MMSE scores of the differences between urban and rural in 2008 and 2018 were decomposed to compare the cognitive ability disparity and contributions of explanatory variables of the two cohort samples. This technology was widely used because it can partition the gap in an outcome variable between two groups into an explained component and an unexplained component (19, 20). Simultaneously, results from this model provide contributions of each explanatory variable to two groups' differentials. In this study, rural-urban disparity in the MMSE scores of older adults in 2008 and 2018 using the O–B decomposition for liner regression models can be written as follows:


(1) $$\begin{array}{l} \begin{array}{ccc} {\bar{Y}}_{t}^{urban} & - & {\bar{Y}}_{t}^{rural}={\bar{X}}_{t}^{rural}({\hat{\beta }}_{t}^{urban}-{\hat{\beta }}_{t}^{rural}) \end{array}\\ \;\begin{array}{cc} + & {\hat{\beta }}_{t}^{rural}({\bar{X}}_{t}^{urban}-{\bar{X}}_{t}^{rural}). \end{array} \end{array}$$

Where ${\bar{Y}}_{t}^{urban}-{\bar{Y}}_{t}^{rural}$ is the disparity of MMSE scores between urban and rural older adults. ${\hat{\beta }}_{t}^{urban}$ and ${\hat{\beta }}_{t}^{rural}$ represent the regression coefficients of the explanatory variables in urban and rural groups, respectively. ${\bar{X}}_{t}^{urban}$ and ${\bar{X}}_{t}^{rural}$ are the corresponding covariate means of the explanatory variables. Owing to the two cohort samples used in this study, t must be assigned to 2008 and 2018 for separate calculation. The first term in Equation (1) is the unexplained component, whereas the second term indicates the explained component.

### Step Two: JMP Decomposition

To further decompose the changes in rural-urban disparity in the MMSE scores of older adults over a 10-year period, JMP decomposition was adopted. Unlike O–B decomposition, this method allows us to estimate the dynamic differences in an unobserved component. By applying this decomposition technique, the changes in the differences in the cognitive function of the older adults between urban and rural over time can be divided into two parts: changes in characteristics and coefficient effects (21).

The change in the cognitive performance gap over time can be written as:


(2) $$\begin{array}{l} D_{2018}-D_{2008}\\ \;=\;\left[\left(X_{urban2018}-X_{urban2008}\right)-\left(X_{rural2018}-X_{rural2008}\right)\right]\\ \;\beta_{2018}+\left(X_{urban2008}-X_{rural2008}\right)\left(\beta_{2018}-\beta_{2008}\right)\\ \;+\;\left[\left(\theta_{urban2018}-\theta_{rural2018}\right)-\left(\theta_{urban2008}-\theta_{rural2008}\right)\right]\\ \delta_{2018}\;+\;(\theta_{urban2008}-\theta_{rural2008})(\delta_{2018}-\delta_{2008}). \end{array}$$

Where “[(*X*_*urban*2018_ − *X*_*urban*2008_) − (*X*_*rural*2018_ − *X*_*rural*2008_)] β_2018_” reflects the changes from 2008 to 2018 in the MMSE score differentials, attributable to differences in the endowments of characteristics between urban and rural (called ‘observed X's/quantity effect'). The term “(*X*_*urban*2008_ − *X*_*rural*2008_)(β_2018_ − β_2008_)” is the “observed price effect,” reflecting changes in responses of the MMSE scores to these observed characteristics differential between urban and rural older adults. The term “[(θ_*urban*2018_ − θ_*rural*2018_) − (θ_*urban*2008_ − θ_*rural*2008_)]δ_2018_” represents the contribution of changes in the rural position in the urban residual distribution and is referred to as the “gap effect.” It measures the gap effect or changes in the levels of the unobservable characteristics. The term “(θ_*urban*2008_ − θ_*rural*2008_)(δ_2018_ − δ_2008_)” is the “unobserved prices effect.” It measures the changes in the rural-urban disparity resulting from the widening distribution of urban MMSE scores of residuals, whilst holding constant the rural-urban gap in unmeasured skills (22).

Statistical analyses were performed using STATA 14.0. A *p*-value of <0.05 was considered statistically significant.

## Results

### Rural-Urban Difference in MMSE Scores and Explanatory Variables in 2008 and 2018

The differences in outcome and explanatory variables between urban and rural in 2008 and 2018 were tested in Table 1. The urban older adults had a better cognitive performance than that of their rural counterparts in the two cohort samples (*p* < 0.001). In addition, urban and rural older adults significantly differed in almost all explanatory variables, particularly in socioeconomic status. For example, employment status and self-rated economic status of urban and rural older adults in the two cohort samples were obviously different (*p* < 0.001). However, age (*p* = 0.816), access to medical services during childhood (*p* = 0.953) and living arrangement (*p* = 0.224) were not significantly different between urban and rural older adults in the 2018 wave.

**Table 1:** Rural-urban difference in MMSE scores and explanatory variables in 2008 and 2018.

**Variables**	**2008 (*****n*** **=** **14,628)**			**2018 (*****n*** **=** **10,311)**		
**(l0ptr0pt)2-4(l0ptr0pt)5-7**	**Urban (*n* = 5,675)**	**Rural (*n* = 8,953)**	P^*^	**Urban(*n* = 5,879)**	**Rural (*n* = 4,432)**	***P***
MMSE (SD)	16.15 (7.52)	15.24 (7.92)	<0.001	19.12 (7.84)	18.29 (7.65)	<0.001
**Gender**			0.007			<0.001
Male (%)	2,516 (44.33)	3,767 (42.08)		2,648 (45.04)	1,817 (41.00)	
Female (%)	3,159 (55.67)	5,186 (57.92)		3,231 (54.96)	2,615 (59.00)	
**Age (year)**			<0.001			0.816
65-75 (%)	1,222 (21.53)	1,749 (19.54)		1,582 (26.91)	1,179 (26.60)	
76-85 (%)	1,167 (20.56)	1,994 (22.27)		1,514 (25.75)	1,142 (25.77)	
86-95 (%)	1,862 (32.81)	2,803 (31.31)		1,386 (23.58)	1,024 (23.10)	
≥96 (%)	1,424 (25.09)	2,407 (26.88)		1,397 (23.76)	1,087 (24.53)	
**Marital status**			<0.001			<0.001
Married and living with spouse (%)	1,886 (33.23)	2,655 (29.65)		2,527 (42.98)	1,730 (39.03)	
Separated/divorced/never married (%)	1,24 (2.19)	258 (2.88)		157 (2.67)	112 (2.53)	
Widowed (%)	3,665 (64.58)	6,040 (67.46)		3,195 (54.35)	2,590 (58.44)	
**Homeownership**			<0.001			<0.001
Own (%)	4,767 (84.00)	8,696 (97.13)		4932 (83.89)	4243 (95.74)	
Not own (%)	908 (16.00)	255 (2.85)		947 (16.11)	189 (4.26)	
**Employment status**			<0.001			<0.001
Not working (%)	1,577 (27.79)	379 (4.23)		2,074 (35.28)	278 (6.27)	
Working (%)	4,098 (72.21)	8,577 (95.80)		3,805 (64.72)	4,154 (93.73)	
Pension			<0.001			<0.001
No (%)	3,573 (62.96)	8,425 (94.10)		3,431 (58.36)	4,070 (91.83)	
Yes (%)	2,102 (37.04)	528 (5.90)		2,448 (41.64)	362 (8.17)	
**Economic status**			<0.001			<0.001
Rich (%)	909 (16.02)	1,014 (11.33)		1,303 (22.16)	675 (15.23)	
Fair (%)	4,045 (71.28)	6,000 (67.02)		4,095 (69.65)	3,190 (71.98)	
Poor (%)	721 (12.70)	1,939 (21.66)		481 (8.18)	567 (12.79)	
**Exercise**			<0.001			<0.001
Yes (%)	2,242 (39.51)	1,800 (20.10)		2,188 (37.22)	1,081 (24.39)	
No (%)	3,433 (60.49)	7,153 (79.90)		3,691 (62.78)	3,351 (75.61)	
**Number of trips**			<0.001			<0.001
0 times (%)	5,215 (91.89)	8,714 (97.33)		4,842 (82.36)	4,083 (92.13)	
≥1 times (%)	460 (8.11)	239 (2.67)		1,037 (17.64)	349 (7.87)	
**Number of social services**			<0.001			0.004
0 (%)	3,832 (67.52)	6,799 (75.94)		2,085 (35.47)	1,702 (38.40)	
1 (%)	746 (13.15)	1,264 (14.12)		1,109 (18.86)	839 (18.93)	
≥2	1,097 (19.33)	891 (9.95)		2,685 (45.67)	1,891 (42.67)	
**Access to medical services in childhood**			<0.001			0.953
Yes (%)	2,308 (40.67)	2,544 (28.42)		510 (8.67)	383 (8.64)	
No (%)	3,367 (59.33)	6,409 (71.58)		5,369 (91.33)	4,049 (91.36)	
**Hunger in childhood**			<0.001			<0.001
Yes (%)	3,761 (66.27)	7,197 (80.39)		3,936 (66.95)	3,538 (79.83)	
No (%)	1,914 (33.73)	1,756 (19.61)		1943 (33.05)	894 (20.17)	
**Living arrangement**			0.009			0.224
With household member (%)	4,824 (85.00)	7,441 (83.11)		4,884 (83.08)	3,625 (81.79)	
Alone (%)	686 (12.09)	1,399 (15.62)		788 (13.40)	634 (14.31)	
In an institution (%)	165 (2.91)	113 (1.26)		207 (3.52)	173 (3.90)	
**Years of schooling**			<0.001			<0.001
0 (%)	3,014 (53.11)	6,150 (68.69)		2,469 (42.00)	2,548 (57.49)	
1-5 (%)	1,332 (23.47)	1,842 (26.49)		1,290 (21.94)	1,044 (23.56)	
≥6 (%)	1,329 (23.42)	961 (10.73)		2,120 (36.06)	840 (18.95)	
**Amount of social security**			<0.001			<0.001
0 (%)	1,370 (24.14)	2,017 (22.52)		359 (6.11)	313 (7.06)	
1-2 (%)	3,757 (66.20)	6,503 (72.63)		5,070 (86.24)	3,989 (90.00)	
≥3 (%)	548 (9.66)	433 (5.00)		450 (7.65)	130 (2.93)	

* *t-test for continuous variable and chi-square test for categorical variables were used to calculate p-value*.

### Urban-Rural Disparity in the Cognitive Performance of Older Adults in 2008 and 2018: O–B Decomposition

Table 2 reports that cognitive ability differences between urban and rural older adults were significant, whereas this gap slightly narrowed from 2008 to 2018. A detailed decomposition analysis revealed that the differences in age, self-rated economic status, regular exercise and years of schooling account greatly for this gap.

**Table 2:** O-B decomposition of the urban-rural disparity in cognitive performance in 2008 and 2018.

	**2008**		**2018**	
	**Coefficient**	**Contribution (%)**	**Coefficient**	**Contribution (%)**
Difference	0.904^**^	100.00	0.833^**^	100
Explained	0.954^**^	105.00	0.681^**^	81.75
Unexplained	−0.050	−5.00	0.152	18.25
**Gender (ref**. **=** **male)**				
Female	0.022^;*^	2.31	0.020^;*^	2.94
**Age (ref**. **=** **65-75)**				
76-85	0.021^;*^	2.20	0.001	0.15
86-95	−0.067	−7.02	−0.018	−2.64
≥96	0.157^;*^	16.46	0.069	10.13
**Marital status (ref**. **=** **married and living with spouse)**				
Separated/divorced/ never married	0.003	0.31	−0.001	−0.15
Widowed	0.028^**^	2.94	0.042^**^	6.17
**Homeownership (ref**. **=** **own)**				
Not own	−0.022	−2.31	−0.071^;*^	−10.43
**Employment status (ref**. **=** **not working)**				
Working	−0.087	−9.12	−0.089	−13.07
**Pension (ref**. **=** **no)**				
Yes	0.078	8.18	0.039	5.73
**Economic status (ref.=** **rich)**				
Fair	−0.021^;*^	−2.20	0.019^;*^	2.79
Poor	0.166^**^	17.40	0.109^**^	16.01
**Exercise (ref**. **=** **Yes)**				
No	0.303^**^	31.76	0.150^**^	22.03
**Number of trips (ref**. **=** **0 times)**				
≥1 times	0.054^**^	5.66	0.035^;*^	5.14
**Number of social services (ref**. **=** **0)**				
1	0.003	0.31	0.001	0.15
≥2	0.061^**^	6.39	0.004	0.59
**Access to medical services in childhood (ref**. **=** **yes)**				
No	0.045^;*^	4.72	0.001	0.15
**Hunger in childhood (ref**. **=** **yes)**				
No	0.071^**^	7.44	0.079^**^	11.60
**Living arrangements (ref**. **=** **With household member)**				
Alone	−0.027^;*^	−2.83	−0.012	−1.76
In an institution	−0.003	−0.31	−0.003	−0.44
**Years of schooling (ref**. **=** **0)**				
1-5	0.037^**^	3.88	−0.033	−4.85
≥6	0.182^**^	19.08	0.327^**^	48.02
**Amount of social security (ref**. **=** **0)**				
1-2	−0.025^;*^	−2.62	−0.012	−1.76
≥3	−0.026^;*^	−2.73	0.024	3.52

* *p < 0.05*;

** *p < 0.001*.

Comparing the two cohort samples, contributions of age (aged over 95) and exercise decreased from 16.46 and 31.76% to 10.13 and 22.03% in 2008 and 2018, respectively, whereas years of schooling (over 5 years) made a greatly increased contribution in this period (19.08-48.02%). In addition to the above variables, other variables explained the cognitive ability disparity between urban and rural older adults to a certain degree. For example, the contributions of experience of hunger in explaining urban-rural disparity accounted for 7.44 and 11.60% in the two cohort samples.

### Changes in Urban-Rural Disparity in the Cognitive Performance of the Older Adults Between 2008 and 2018: JMP Decomposition

A narrowing of ~0.071 in the urban-rural disparity in the cognitive ability of older adults was found during the period 2008-2018 as whole (Table 3). After decomposition, observed and unobserved components have countervailing effects on the changes in urban-rural disparity. That is, observed components largely served to narrow urban-rural disparity in cognitive performance of the older people, but it was hampered by those in the unobserved components, which resulted in a small reduction in the cognitive performance gap between urban and rural older adults. Amongst the observed components, contribution of quantity effects (65.21%) was slightly higher than that of price effects (46.84%).

**Table 3:** JMP decomposition of the change in urban-rural disparity in cognitive performance.

	**Coefficient**	**Contribution (%)**
Changes in rural-urban disparity	−0.071	100
Difference in predicted gap	−0.365	514.08
Quantity effect	−0.238	65.21
Price effect	−0.171	46.84
Quantity effect * price effect	0.044	12.05
Difference in residual gap	0.295	−415.49

In terms of quantity effects, the reduced disparity in the proportion of people who aged over 95 (35.71%), had self-rated poor economic status (33.19%) and had no exercise (56.72%) between urban and rural were mainly responsible for the reduced cognitive ability differentials (Table 4). However, old people with other characteristics distributed more unevenly between urban and rural from 2008 to 2018, which hampered the narrowed cognitive ability gap. For example, the changes in the share of pension coverage, employment, going to at least one trip and the benefits of at least three social securities between urban and rural older adults contributed −27.31, −16.39, −16.39, and −23.11% to the narrowed cognitive function disparity.

**Table 4:** Quantity and priced effects of explanatory variables on the change in urban-rural disparity in cognitive performance.

	**Quantity effect**		**Priced effect**	
	**Coefficient**	**Contribution (%)**	**Coefficient**	**Contribution (%)**
**Gender (ref**. **=** **male)**				
Female	0.015	−6.30	−0.011	6.43
**Age (ref**. **=** **65-75)**				
76-85	−0.016	6.72	−0.003	1.75
86-95	−0.014	5.88	0.007	−4.09
≥96	−0.085	35.71	0.008	−4.68
**Marital status (ref**. **=** **married and living with spouse)**				
Separated/divorced/ never married	−0.008	3.36	−0.007	4.09
Widowed	0.009	−3.78	−0.002	1.17
**Homeownership (ref**. **=** **own)**				
Not own	−0.003	1.26	−0.094	54.97
**Employment status (ref**. **=** **not working)**				
Working	0.039	−16.39	−0.006	3.51
**Pension (ref**. **=** **no)**				
Yes	0.065	−27.31	−0.016	9.36
**Economic status (ref**. **=** **rich)**				
Fair	0.001	−0.42	−0.012	7.02
Poor	−0.079	33.19	0.019	−11.11
**Exercise (ref**. **=** **Yes)**				
No	−0.135	56.72	−0.144	84.21
**Number of trips (ref**. **=** **0 times)**				
≥1 times	0.039	−16.39	−0.032	18.71
**Number of social services (ref**. **=** **0)**				
1	−0.028	11.76	−0.018	10.52
≥2	−0.018	7.56	−0.012	7.02
**Access to medical services in childhood (ref**. **=** **yes)**				
No	−0.027	11.34	−0.019	11.11
**Hunger in childhood (ref**. **=** **yes)**				
No	−0.003	1.26	0.046	−26.90
**Living arrangements (ref**. **=** **with household member)**				
Alone	0.011	−4.62	0.003	−1.75
In an institution	−0.022	9.24	0.002	−1.17
**Years of schooling (ref**. **=** **0)**				
1-5	−0.022	9.24	0.037	−21.64
≥6	0.005	−2.10	0.095	−55.56
**Amount of social security (ref**. **=** **0)**				
1-2	−0.015	6.30	−0.024	14.04
≥3	0.055	−23.11	0.012	−7.02

Priced effects revealed that the effects of urban and rural differentials in homeownership (54.97%), regular exercise (84.21%) and number of trips (18.71%) on cognitive function disparity weakened over time. However, the effects of other explanatory variables, such as hunger in childhood (−26.90%) and years of schooling (−77.20%) strengthened during the 2008–2018 period, thereby worsening the urban and rural gap of cognitive performance.

## Discussion

This study reveals a reduction in the cognitive performance of older adults' differentials between urban and rural older adults in China from 2008 to 2018. This change is attributable to the quantity and priced effects of various explanatory factors.

The change in the proportion of people ≥ 96 years between urban and rural significantly contributed to the decline in cognitive performance disparity. The underlying reason may be because the considerable investment in the rural health system in recent 10 years has increased the life expectancy of the rural population in China (23). These efforts balanced the share of older adults between urban and rural areas. Accordingly, age-related cognitive disorder disparity between urban and rural older adults are expected to be reduced as well.

Interestingly, an effect of homeownership gap on cognitive ability disparity in urban and rural seemingly decreased in the 2018 wave. The association of homeownership with cognitive health is mainly due to the mediation effects of the perceived sense of control, community trust and residential stability (24, 25). However, China's accelerated urbanization in the past period resulted in the increased number of old people who migrate from rural to urban with their children (26). Frequent mobility of residence reflects that owning a house at present does not mean an increased sense of control and stability for senior residents. Consequently, the effect of homeownership on the cognitive ability of older adults decreased accordingly in 2018 comparing with that in 2008.

Another encouraging finding is that the reduction in self-rated poor economic status amongst rural older adults positively contributed to the narrowed cognitive disorder differential. The rapid socioeconomic development in China over the past few decades significantly increased income growth in rural regions, and the establishment of a universal medical insurance system also decreased the out-of-pocket payment ratio for medical care (27, 28). Consequently, financial hardship was substantially reduced when rural older adults sought medical services, which further decreased exposure to the risk factors of cognitive impairment owing to timely treatments.

Our findings also reveal that an increased share of rural older adults who exercise regularly reduced the cognitive function inequality greatly between urban and rural. These findings were supported by previous studies reporting physical exercises can increase production of neurotrophic factors and cerebral blood flow, as well as further prevent degeneration of the brain's cognitive function with aging (29, 30). Furthermore, given that the Chinese central government increased investment in the basic rural healthcare infrastructure, and health education services were widely provided in rural areas in recent years, more older people are encouraged to take physical exercises, thus delaying the damage of cognitive decline. Moreover, the finding that price effect of trips declined over time might reflect other recreational activities in rural regions, such as Chinese chess and square dancing, which are often organized to improve the well-being of rural older adults, thereby offsetting the impact of the urban-rural inequality in travel participation.

A gap in proportion to the older adults who still worked during retirement age between urban and rural exacerbated the widening cognitive function disparity. A potential explanation can be attributed to the urban-rural gap in social security and welfare benefits. On the one hand, social security coverage in rural areas is much lower than that in urban areas, even though the Chinese government proposed the establishment of a rural endowment insurance system in 2009. On the other hand, the level of social security and welfare benefits rural older adults can receive is also quite limited because of the great urban-rural disparity in economic development (31, 32). Resultantly, rural older adults must work to earn income and thus endure more pressure from their jobs, which create an adverse effect on cognitive ability. Similarly, the quantity effects of pension and amount of social security also revealed that an unbalanced distribution of social security between urban and rural was expanding over time, which further caused the expansion of the cognitive function differentials. Prior studies have proved that expansions in social security and welfare benefits have important protective effects on mental illnesses, especially for disadvantaged groups, by providing additional income to increase financial security and reduce social status anxiety (33, 34). A large room exists for bridging the cognitive performance gap by improving social security coverage and welfare benefits for rural older adults.

Individual experiences in childhood can influence health and well-being in the late periods of life. Exposure to fetal malnutrition has considerable and long-lasting impacts on physical health and cognitive abilities (35). “Cognitive reserve” theory suggests that early education can delay manifestation of dementia symptoms in much older adults by allowing the brain to better cope with damages (36). In this study, the participants in our analysis were born before 1953. They endured the Great Famine and the Cultural Revolution in China, which led to malnutrition and poor education, especially in the rural group. Therefore, this study finds that rural older adults who suffered from hunger and poor education at a young age performed worse in cognitive ability, subsequently widening the urban-rural difference. Moreover, this impact intensifies over time. An implication from this evidence supported policies and programs to improve the nutritional status and education level for children at an early stage, especially in China's underdeveloped rural areas.

This study holds some limitations the must be acknowledged. Firstly, community- and provincial-level variables were not used to explore their contributions to the changes in the cognitive performance differences between urban older adults and rural older adults due to limited data, even though a comprehensive set of individual level variables was included on the basis of Lund's conceptual framework. Secondly, survival bias may exist, because the CLHLS focuses on the long lived, and the survey may exclude people who did not live long lives. Thus, a healthy sample can be presented, resulting in an underestimated cognitive impairment. Thirdly, data in CLHLS were collected by self-report. Recall bias and measurement bias should be paid an attention.

## Conclusion

Although the urban-rural disparity in the cognitive performance of older adults in China narrowed from 2008 to 2018, it remains a significant problem. The rapid economic development and reform of the rural health system significantly contributed to the narrowed gap. However, then unbalanced distribution in social security and welfare between urban and rural regions resulted in an expanding trend in the urban-rural inequality of cognitive function. Additionally, future policy interventions must highlight rural older adults who suffered from hunger and limited education during childhood, because the negative effects of these hardships on cognitive function intensify late in life.

## Data Availability Statement

The original contributions presented in the study are included in the article/Supplementary Material, further inquiries can be directed to the corresponding author/s.

## Ethics Statement

The studies involving human participants were reviewed and approved by the Institutional Review Board, Duke University (Pro00062871), and the Biomedical Ethics Committee, Peking University (IRB00001052–13074). Written informed consent to participate in this study was provided by the participants' legal guardian/next of kin.

## Author Contributions

TZ was responsible for the study design, implementation, and writing. BL collected and analyzed the data and reviewed and edited original draft. XW revised and critically commented the manuscript. All authors made significant contributions to this study and have read and approved the final manuscript.

## Funding

This work was supported by National Natural Science Foundation of China (grant number: 71974050) and the Scientific Research Foundation for Scholars of HZNU (grant number: 4265C50221204120).

## Conflict of Interest

The authors declare that the research was conducted in the absence of any commercial or financial relationships that could be construed as a potential conflict of interest.

## Publisher's Note

All claims expressed in this article are solely those of the authors and do not necessarily represent those of their affiliated organizations, or those of the publisher, the editors and the reviewers. Any product that may be evaluated in this article, or claim that may be made by its manufacturer, is not guaranteed or endorsed by the publisher.
