# Supplementary material for: Urban-Rural Disparity in Cognitive Performance Among Older Chinese Adults: Explaining the Changes From 2008 to 2018
Source: Front Public Health. 2022 Mar 23;10:843608. doi: 10.3389/fpubh.2022.843608 (PMC8984104; doi:10.3389/fpubh.2022.843608)
Supplement: Supplementary file 1 [file Table_1.DOCX]

**Supplemental table 1 Explanatory variables and their measurements by five domains**

| **Domain** | **Variables** | **Measurements** |
| --- | --- | --- |
| Demographic (The specific demographic characteristics of populations that convey risk for, or protection from,  mental illness) | Gender | Sex reported by respondents |
|  | Age | Age reported by respondents |
|  | Marital status | Current marital status reported by respondents |
| Economic (Factors relating to the production, consumption, and transfer of wealth that convey risk for, or protection from, mental  illness) | Homeownership | Do you own the property rights of the house you currently live in |
|  | Employment status | Are you retired now? If you have retired, are you still engaged in paid jobs now? |
|  | Pension | Do you have a pension for retirement |
|  | Self-rated economic status | How do you rate your economic status compared with others in your local area? |
| Neighborhood (Characteristics of a community that convey risk for, or protection from, mental illness, over and above what is attributable to the individual characteristics of community members) | Exercise | Do you exercise regularly near where you live at present |
|  | Number of trips organized | How many tours organized have you made in the past two years |
|  | Number of social services in community | What kind of social services are available in your community (personal daily care services, home visits, psychological consulting, daily shopping, social and recreation activities, legal aid, health education, neighboring relations and others) |
| Environmental events (Serious disruptions of the functioning of a community that exceed its ability to cope by use of its own resources and convey risk for mental illness) | Access to medical services in childhood | Could you get adequate medical service when you were sick in childhood |
|  | Hunger in childhood | Did you frequently go to bed hungry as a child |
| Social and cultural (ways in which the organization of society, social interactions, and relationships affect risk of, and protection from,  mental illness) | Living arrangement | Co-residence reported by respondents |
|  | Years of schooling | How many years did you attend school |
|  | Number of social security | Do you have following social security and commercialized insurances at present (None, retirement pension, public old-age insurance, commercialized old age insurance, public free medical services, medical insurance for urban workers and residents, the new rural cooperative medical insurance, commercial medical insurance and other) |
